# 

**Published:** 1892-11-05

**Authors:** 


CONTENTS.

| | PAGE |
|---|---|
| A Danger to be Averted | 81 |
| Passing Topics.—Personalties | 82 |
| Diet in Disease. By Mrs. Ernest Hart. V.—Restoratives: Tea | 83 |
| Medical History from the Earliest Times. By E. T. Withington, M.A., M.B.Oxon. XXX.—Arabic Medicine—(3) Haly Abbas and Avicenna (*continued*) | 85 |
| Contemporary Theories and Research. The Vibrating Helmet—The Birthplace of Cholera | 86 |
| Annotations.—Converted Clowns as Hospital Chaplains—Medical Peers—Heart Disease | 87 |
| Notes and News | 88 |
| Scraps and Gleanings | 96 |
| The Hospital Clinic— | |
| Middlesex Hospital.—Treatment of Enteric Fever | 89 |
| Hull Royal Infirmary.—Treatment of Fractures of the Leg | 90 |
| Royal Infirmary, Edinburgh—The Treatment of Chronic Suppuration of the Middle Ear | 91 |
| The Morality of Vivisection. By Prof. Victor Horsley, F.R.S. | 92 |
| Vivisection: An Appeal to Reason | 92 |
| The Institutional Workshop— | |
| Within the Hospitals.—Glasgow and its Medical Institutions | 93 |
| Practical Departments.—II. Children's Cots | 94 |
| Colonial Hospitals.—Hospitals in New Zealand | 95 |
| The Student of Medicine.—Appointments | |

EXTRA SUPPLEMENT.—THE NURSING MIRROR.

| | |
|---|---|
| En Passant.—Leeds District Nurses—Testimonials and Presents—Incurable Children—Stockton and Thornaby—The Guildford Institute—Lowestoft Provident Nurses' Fund—Hull District Nurses—Dundee District Nurses—The Sarah Acland Home, Oxford | xxxi |
| Lectures for Asylum Attendants. By William Harding, M.B.—VI. Some Important Points: Excretions, &c. | xxxii |
| Mary Wardell Convalescent Home for Scarlet Fever | xxxii |
| A Nurse's View of Christmas Decorations | xxxiii |
| Our Indian Letter | xxxiv |
| Everybody's Opinion.—Home for an Incurable—Uniforms as Advertisements—Assistants' Manners and Nurses' Fees | xxxv |
| Appointments | xxxv |
| For Reading to the Sick.—Reaping | xxxv |
| Off Duty.—Glasgow Nurses "Off Duty." | xxxvi |
| Notes and Queries | xxxvi |

